# Isocitrate Dehydrogenase (IDH)-Wildtype Glioblastoma With an Unusual EWSR1::RAD51B Gene Fusion: A Case Report and Review of the Literature

**DOI:** 10.7759/cureus.115029

**Published:** 2026-08-23

**Authors:** Sahar Al-Mustafa, Mustafa Al-Kawaaz, Litty Paul, Adelisa Franchitti, Kenneth M Aldape, Eyas M Hattab

**Affiliations:** 1 Department of Pathology and Laboratory Medicine, University of Louisville, Louisville, USA; 2 Department of Pathology and Laboratory Medicine, King Hussein Cancer Center, Amman, JOR; 3 Next Generation Sequencing Laboratory, University of Louisville Hospital, Louisville, USA; 4 Department of Pathology and Laboratory Medicine, Mayo Clinic, Rochester, USA; 5 Department of Pathology, University of Louisville School of Medicine, Louisville, USA

**Keywords:** dna methylation profiling, ewsr1 fusion, glioblastoma, idh‑wildtype, molecular pathology, rad51b

## Abstract

Glioblastoma (GBM) is the most common primary malignant neoplasm of the central nervous system in adults. GBM is defined as an isocitrate dehydrogenase (IDH)-wildtype diffuse astrocytic glioma with characteristic histologic and/or molecular features. The expanding use of integrated histologic, molecular, and epigenetic diagnostics has revealed increasing biological heterogeneity within this category. We report a case of a 73-year-old man with GBM, IDH-wildtype, harboring a novel EWSR1::RAD51B gene fusion identified by next-generation sequencing (NGS). Magnetic resonance imaging revealed a heterogeneous, predominantly peripherally enhancing mass involving the left temporal, parietal, and occipital lobes. Histologic examination demonstrated a diffusely infiltrative high-grade glioma with marked nuclear pleomorphism, brisk mitotic activity, microvascular proliferation, and geographic and pseudopalisading necrosis. Immunohistochemistry was supportive of GBM. Molecular studies revealed MGMT promoter methylation and a truncating PTEN mutation (p.Q214*, c.640C>T). In addition, a previously unreported EWSR1::RAD51B gene fusion was identified by NGS. DNA methylation profiling also classified the tumor as GBM, IDH-wildtype, with high confidence. To our knowledge, this is the first reported case of GBM harboring an EWSR1::RAD51B gene fusion. Although the biological significance of this alteration remains unclear, its identification expands the molecular spectrum of GBM and underscores the value of comprehensive genomic and epigenomic profiling in the diagnostic evaluation of diffuse gliomas, particularly when unexpected or atypical molecular findings are encountered.

## Introduction

Glioblastoma (GBM) is the most common primary malignant neoplasm of the central nervous system (CNS) in adults and is associated with a dismal prognosis despite aggressive multimodal therapy. The 2021 WHO classification defines GBM as an IDH-wildtype diffuse astrocytic glioma characterized histologically by necrosis and/or microvascular proliferation [1]. In the absence of these histologic features, specific molecular alterations, including combined whole chromosome 7 gain with chromosome 10 loss (+7/−10), telomerase reverse transcriptase (TERT) promoter mutation, and epidermal growth factor receptor (EGFR) amplification, were considered important molecular hallmarks for GBM diagnosis [2]. However, emerging data suggest that “molecular GBMs” lacking histologic features of GBM may not behave as aggressively as histologically defined tumors, advising caution when encountering solitary molecular alterations [3,4]. The integration of molecular diagnostics has substantially refined glioma classification and enabled more precise diagnostic and prognostic stratification [5,6]. Furthermore, DNA methylation profiling has demonstrated that GBM comprises biologically and prognostically distinct subgroups, supporting its role beyond diagnostic classification alone [7,8].

The EWSR1 (Ewing sarcoma breakpoint region 1) gene is classically associated with recurrent gene fusions in mesenchymal tumors, where it typically functions as part of oncogenic chimeric transcription factors that create new, abnormal proteins that drive cancer by altering gene expression [9,10]. EWSR1 fusion proteins were first recognized as central drivers in Ewing sarcoma and other soft tissue mesenchymal tumors [10,11]. Several distinct CNS tumor entities defined by EWSR1 fusions are now incorporated into the WHO framework, including glioneuronal tumors with EWSR1::PLAGL1 fusions, neuroepithelial tumors with EWSR1::PATZ1 fusions, and intracranial mesenchymal tumors with FET::CREB family fusions [12-17]. To date, however, EWSR1 gene rearrangement has not been reported in conventional IDH-wildtype GBM. EWSR1 can lead to glioma progression and resistance to temozolomide [18]. RAD51 and its human analog, RAD51B, function to catalyze adenosine triphosphate (ATP)-dependent DNA recombination during DNA repair [19-21]. Rearrangements involving RAD51B have been described in certain sarcomas and PEComas, with the notion that tumors harboring RAD51B fusions exhibit heterogeneous phenotypes [22-24].

Here, we describe a case of IDH-wildtype GBM harboring a novel EWSR1::RAD51B gene fusion, representing a previously unrecognized molecular alteration in this tumor. This case highlights the importance of integrated histologic, molecular, and epigenetic diagnostics when encountering unexpected gene fusions in high-grade CNS tumors.

This case report was previously presented as a poster at the 102nd annual meeting of the American Association of Neuropathology (AANP) on June 5, 2026.

As molecular profiling continues to refine the characterization of GBM, reporting rare genetic alterations is important for improving understanding of tumor biology. Given the rarity of the EWSR1::RAD51B fusion in GBM, this case contributes valuable information to the emerging literature on the molecular landscape of this disease and its potential clinical significance.

## Case presentation

Clinical history and imaging

Clinical Presentation

A 73-year-old man with a medical history of hypertension and hyperlipidemia presented in May 2025 with several months of progressive cognitive decline, first noticed in May 2024, short-term memory impairment that had progressed over the preceding four weeks beginning in April 2025, and fatigue. The patient also presented with a four-week history of progressive word-finding difficulty, right-sided hemisensory loss, and mild right-hand clumsiness. Neurological examination revealed mild disorientation and deficits in short-term memory but no focal motor weakness. Based on the patient's presentation, the differential diagnosis included high-grade glioma, primary CNS lymphoma, metastatic brain tumor, subacute cerebral infarction, and other infiltrative or inflammatory lesions of the left cerebral hemisphere, prompting comprehensive radiologic evaluation.

Radiologic Evaluation

**Figure 1:**
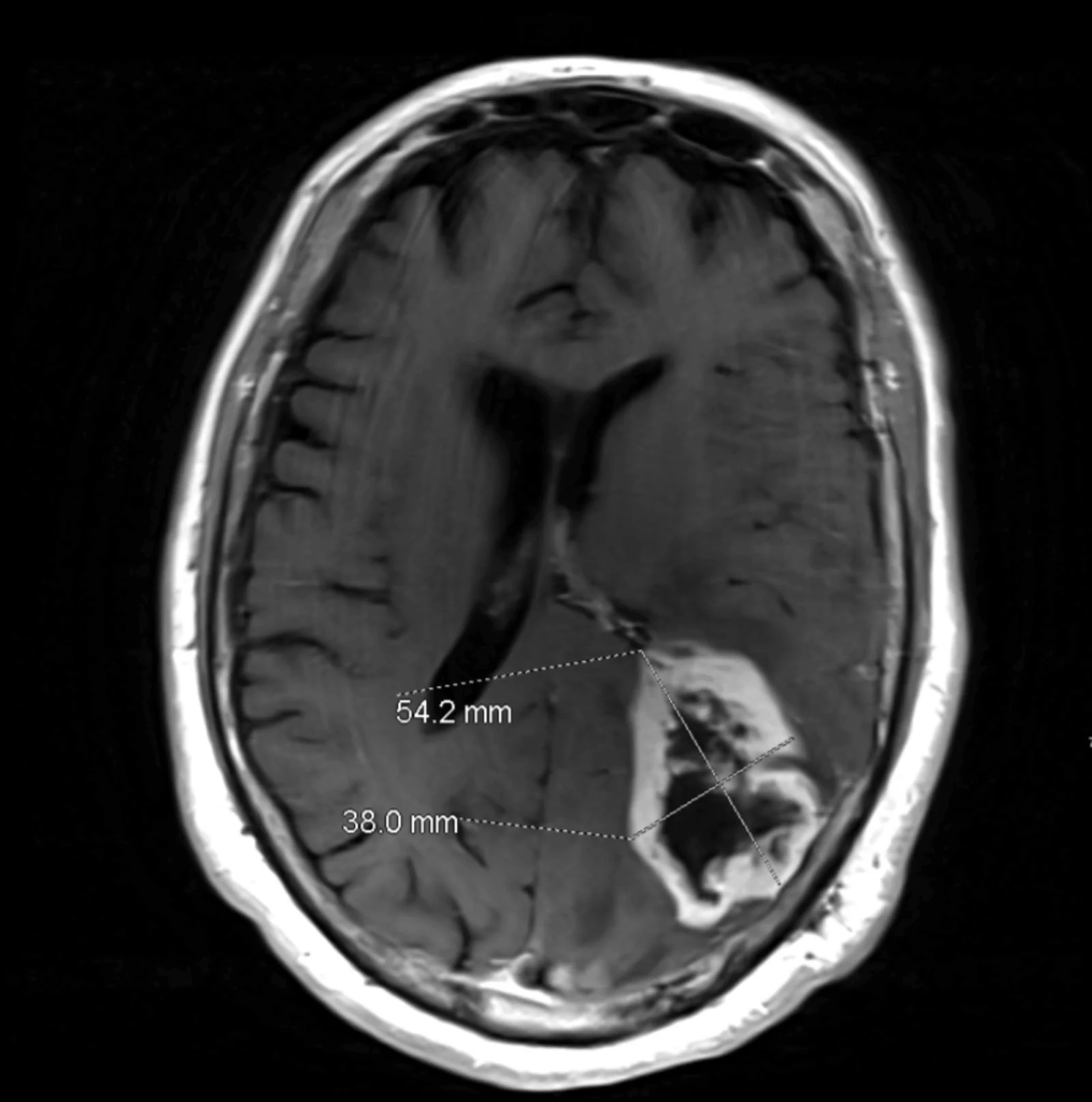
T1/FLAIR post-contrast brain MRI revealed a 5.4-cm heterogeneously enhancing mass in the left temporo-parieto-occipital region, with prominent perilesional edema, mild midline shift (7 mm), and internal areas of nonenhancing necrosis. FLAIR, fluid-attenuated inversion recovery; MRI, magnetic resonance imaging

Magnetic resonance imaging (MRI) (Figure 1) demonstrated a heterogeneous, predominantly peripherally enhancing mass involving the left temporal, parietal, and occipital lobes, measuring approximately 5.4 × 3.8 × 5.0 cm. The lesion showed central necrosis with surrounding vasogenic edema and mass effect on the adjacent parenchyma. The radiologic impression favored a high-grade glioma.

Pathologic findings

Histopathologic Examination

**Figure 2:**
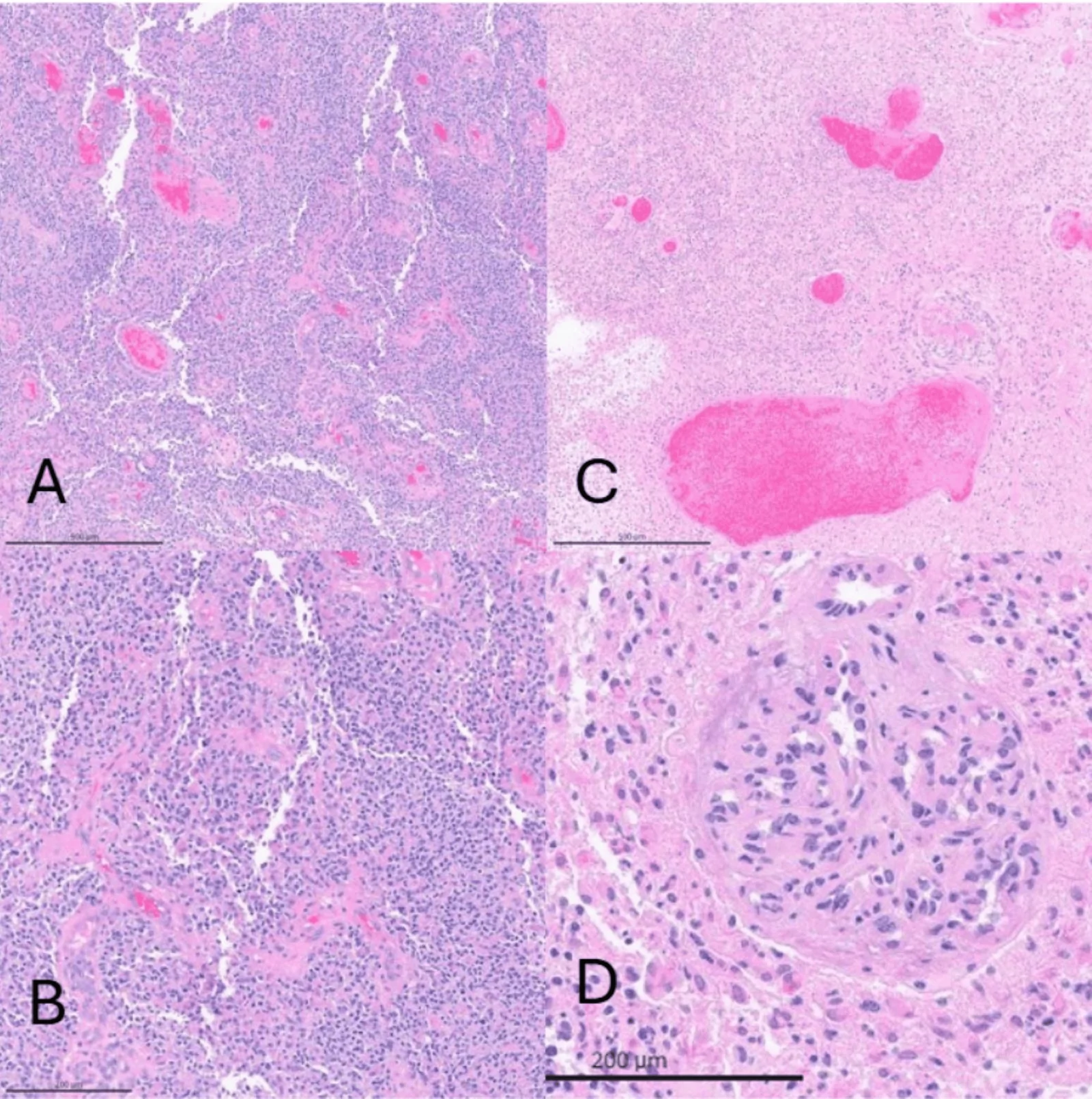
Representative hematoxylin and eosin-stained sections showing a highly cellular astrocytic neoplasm with nuclear pleomorphism (A, 50×; B, 100×), necrosis (C, 100×), and microvascular proliferation (D, 100×).

Permanent sections demonstrated a diffusely infiltrative glial neoplasm involving the cerebral cortex and underlying white matter. The tumor was moderately to highly cellular and composed of atypical astrocytic cells with marked nuclear pleomorphism, hyperchromasia, and frequent multinucleation. Brisk mitotic activity was identified (>20 mitoses per 10 high-power fields in the most active areas). Prominent microvascular proliferation was present, with multilayered endothelial cell tufts and glomeruloid vascular structures. Geographic necrosis with pseudopalisading tumor cells was readily apparent (Figure 2).

**Figure 3:**
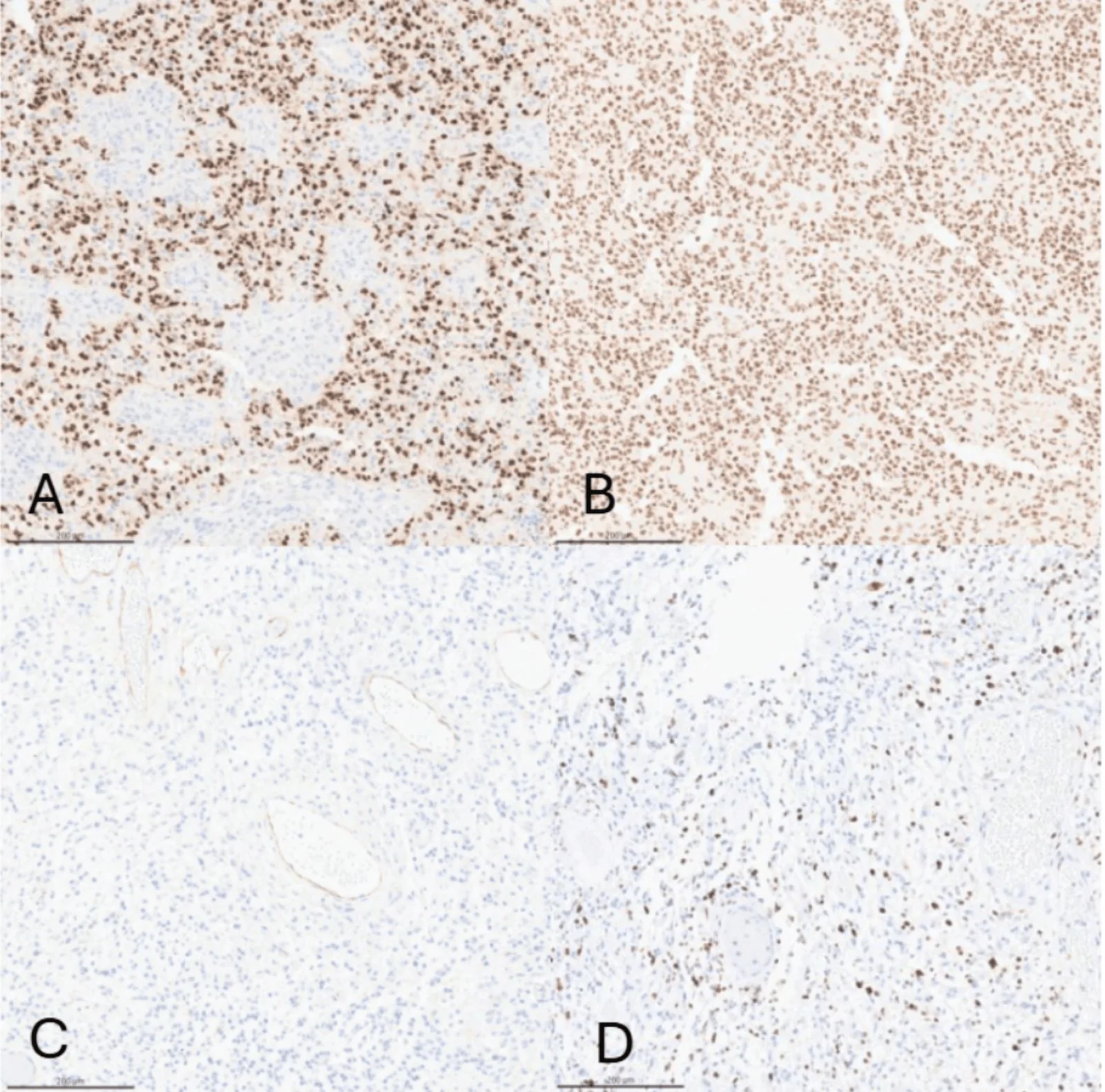
Immunohistochemical stains: A, OLIG2 (100×); B, ATRX (100×); C, IDH1-R132H (100×); D, MIB-1 (Ki-67) (100×).

Immunohistochemical studies revealed strong nuclear positivity for OLIG2 (Figure 3A), supporting a diffuse glioma lineage. IDH1-R132H immunostaining was negative (Figure 3C). Nuclear expression of ATRX was retained (Figure 3B).

The Ki-67 (MIB-1) proliferation index was elevated, reaching approximately 20% in areas of highest proliferative activity, in keeping with a high-grade glioma (Figure 3D).

Molecular Studies

MGMT promoter methylation analysis demonstrated MGMT promoter methylation, suggesting potential sensitivity to alkylating agent chemotherapy.

**Figure 4:**
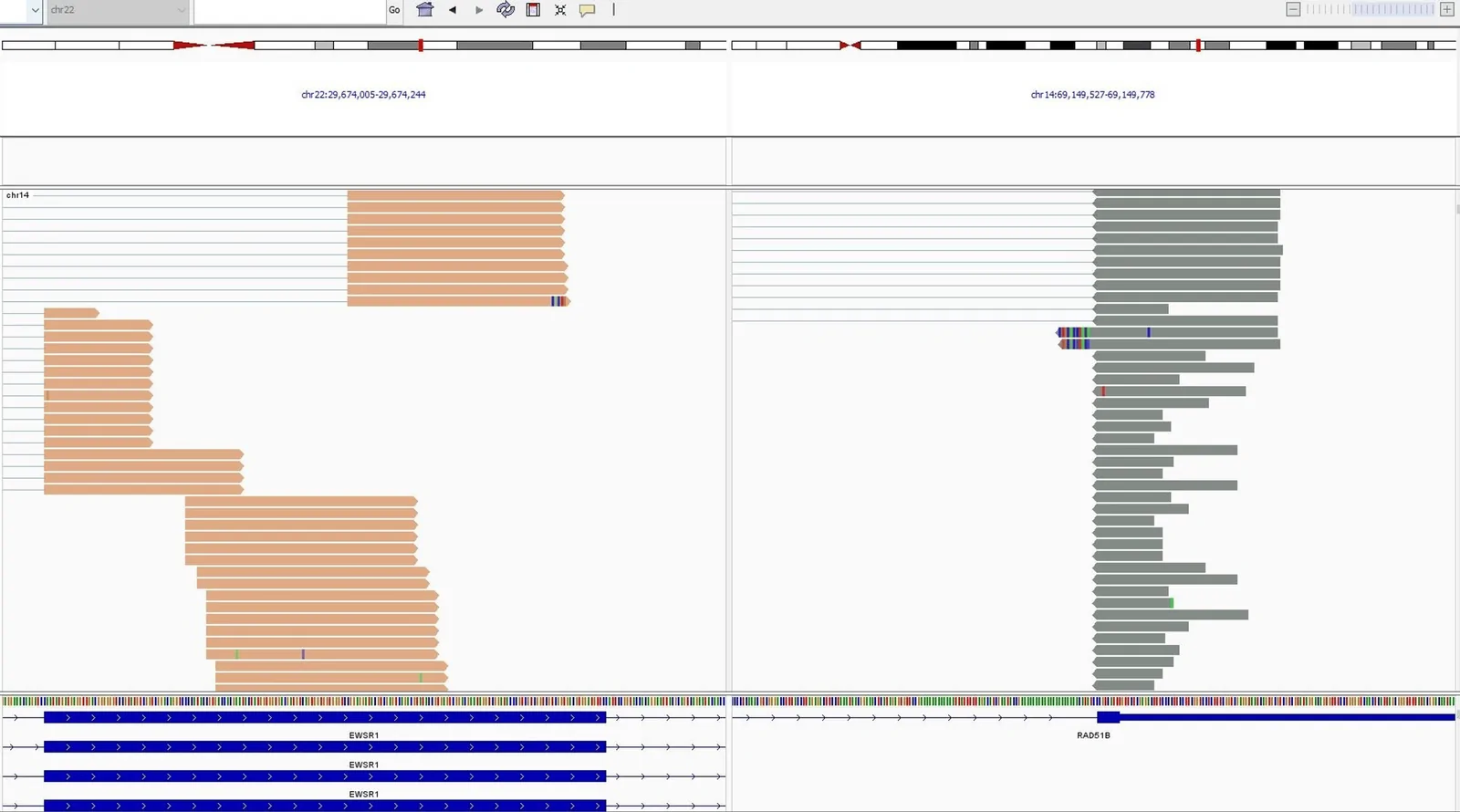
IGV was used to visualize the EWSR1::RAD51B gene fusion. Split-screen view of read alignments from next-generation sequencing of the tumor sample, displaying regions of chromosomes 22 and 14. In this view, alignments are grouped and sorted by the chromosome of the mate. Alignment reads in IGV are color-coded according to the chromosome of the mate. IGV, Integrated Genome Viewer

Targeted next-generation sequencing (NGS) (Illumina TruSight Oncology Comprehensive Assay) identified a truncating PTEN mutation (p.Q214*, c.640C>T) with a variant allele frequency of 76.3%, consistent with a pathogenic alteration involving the PI3K/AKT pathway. In addition, a novel EWSR1::RAD51B gene fusion was detected (Figure 4).

DNA methylation profiling was performed at the National Institutes of Health using a genome-wide methylation array. The tumor was classified as GBM, IDH-wildtype, with a high calibrated score, providing independent epigenetic confirmation of the diagnosis.

Clinical Course

The patient's preoperative Karnofsky Performance Score was 70. Postoperatively, the patient recovered without any major new neurologic deficits. Given his age and extent of resection, a hypofractionated course of involved-field radiotherapy (34 Gy in 10 fractions) was administered concurrently with temozolomide, initially started at a standard dose of 150 mg/m²; however, treatment was poorly tolerated during the first cycle. Consequently, the regimen was modified to 50 mg/m² administered daily for 21 consecutive days, followed by a seven-day treatment-free interval. The patient tolerated the modified regimen well, with minimal treatment-related symptoms and an overall stable clinical condition. Follow-up MRI at seven months showed a stable resection cavity with no new areas of enhancement, consistent with stable disease per Response Assessment in Neuro-Oncology (RANO) criteria [25]. In addition, clinically, the patient is doing well, with no deterioration in motor, language, or cognitive function.

## Discussion

GBM, IDH-wildtype, represents the most common and biologically aggressive diffuse glioma in adults and is characterized by marked molecular heterogeneity [1,5,6]. The 2021 WHO classification emphasizes an integrated diagnostic approach combining histologic and molecular features, recognizing that specific molecular alterations may supersede histologic grade in defining GBM [1,2]. This framework was initially addressed in cIMPACT-NOW update 3, which clarified and qualified the role of molecular criteria in glioma classification, with the recommended designation “diffuse astrocytic glioma, IDH-wildtype, with molecular features of GBM, WHO grade 4” for IDH-wildtype diffuse astrocytomas that lack classic histologic features of GBM but harbor one or more of the following: EGFR amplification, combined whole chromosome 7 gain and chromosome 10 loss (+7/-10), or TERT promoter mutation [3]. More recently, cIMPACT-NOW update 11 further refines these concepts by emphasizing age-dependent considerations and cautioning against upgrading lower-grade diffuse gliomas to GBM solely based on solitary molecular alterations in younger patients, particularly when clinical, radiographic, and histologic features are discordant [4]. This underscores the necessity of integrating clinical context, histopathology, and molecular data when establishing a diagnosis of GBM.

Genomic studies have shown that GBM is characterized by recurrent alterations in receptor tyrosine kinase signaling (e.g., EGFR, PDGFRA), the PI3K/AKT pathway (e.g., PTEN, PIK3CA), cell-cycle regulators (e.g., CDKN2A/B, RB1), and tumor suppressor genes (e.g., TP53) [5,6]. Chromosomal rearrangements and gene fusions are relatively uncommon in GBM compared with other CNS tumor entities; however, rare fusions, most notably involving EGFR, have been documented and may contribute to oncogenesis through aberrant activation of growth-promoting pathways [6].

In the present case, the diagnosis of GBM was supported by classic histopathologic features, including microvascular proliferation and necrosis, in conjunction with IDH-wildtype status and a truncating PTEN mutation. Importantly, DNA methylation-based profiling provided independent confirmation, classifying the tumor as GBM, IDH-wildtype, with high confidence. In recent years, DNA methylation-based classification has emerged as a robust diagnostic modality, particularly in cases with unusual or unexpected molecular findings, and has demonstrated superior reproducibility and diagnostic accuracy compared with histopathology alone [7,8]. 

In this case, an EWSR1 gene fusion was identified by NGS. The EWSR1 gene encodes a multifunctional RNA-binding protein involved in transcriptional regulation, RNA processing, and chromatin remodeling. Oncogenic EWSR1 fusions typically generate chimeric transcription factors that drive tumorigenesis via dysregulated gene expression programs [9,10]. These fusion proteins were first characterized in Ewing sarcoma and other soft tissue tumors [10-12]. In addition, several CNS tumor entities defined by EWSR1 fusions are now recognized within the WHO classification, including glioneuronal tumors with EWSR1::PLAGL1 fusions, neuroepithelial tumors with EWSR1::PATZ1 fusions, and intracranial mesenchymal tumors with FET::CREB family fusions [13-17].

The presence of an EWSR1::RAD51B fusion in GBM challenges the notion that EWSR1 rearrangements are restricted to mesenchymal or glioneuronal lineages. Given the context of an otherwise typical adult IDH-wildtype GBM with characteristic histology, OLIG2 positivity, retained ATRX expression, MGMT promoter methylation, a truncating PTEN mutation, and high-confidence methylation-based classification, the EWSR1::RAD51B fusion may represent a rare, context-dependent alteration. It remains uncertain whether this fusion functions as a primary oncogenic driver or as a passenger event arising from genomic instability. Nonetheless, its identification expands the documented molecular spectrum of GBM. The role of EWSR1 in glioma pathogenesis is not fully understood; however, emerging evidence indicates that EWSR1 may influence glioma biology through mechanisms other than its classical role in fusion oncoproteins. In particular, the EWSR1-regulated circular RNA circNEIL3 has been reported to interact with U2AF2, enhancing the stability of SPI1 mRNA and thereby driving glioma progression and resistance to temozolomide [18]. The RAD51B gene was initially described in yeast and was later identified in humans and mapped to chromosome 14. This gene encodes a protein that catalyzes ATP-dependent DNA recombination, which plays an important role in DNA repair during cell division [19,21]. Fusions involving RAD51B are considered rare in solid tumors, with reported cases occurring most commonly in certain uterine sarcomas and PEComa [22-24]. The role of RAD51B in glioma pathogenesis also remains incompletely understood. In one study, lower levels of RAD51B were associated with glioma progression and susceptibility [26]. Although the biological impact of the EWSR1::RAD51B fusion observed in our case remains unclear, its presence underscores the need for additional studies in larger cohorts to determine whether this alteration defines a distinct molecular subset of GBM or instead represents a rare passenger event. A plausible explanation for this association is that a fusion involving RAD51B may impair DNA repair, leading to genomic instability. This possibility highlights the importance of further advanced molecular investigations, including homologous recombination deficiency (HRD) testing, as identifying HRD in this tumor could have therapeutic implications, particularly regarding the potential use of poly(ADP-ribose) polymerase (PARP) inhibitors [27].

The primary limitation of this report is its single-case nature; the clinical and biological relevance of this novel fusion cannot be generalized without larger cohort studies.

## Conclusions

We describe a case of GBM, IDH-wildtype, with a novel EWSR1::RAD51B fusion not previously reported in this tumor entity. This case broadens the known molecular landscape of GBM and underscores the utility of integrated molecular profiling in characterizing rare genomic alterations.
